# Medicine

**Published:** 1908-03

**Authors:** J. R. Charles


### Progress of the Medical Sciences.

### MEDICINE.

**Hyperthyroidism.**—Typical Graves's disease is diagnosed without difficulty. Many cases are, however, met with clinically which may be regarded as atypical, to which the term hyperthyroidism is applied. Cases are now frequently regarded as belonging to this category which formerly were classed as neurasthenia, hysteria, functional heart disease, &c. A. R. Elliott[1] regards the question of the pathogenesis of Graves's disease settled in favour of the theory of Möbius, viz. that a perverted over-activity of the thyroid is the essential feature. In support of this view he quotes the results of surgical treatment in Graves's disease, and the experimental evidence which has been obtained from thyroid feeding.

The exact rôle played by the thyroid in physiological metabolism is still largely a matter of conjecture. The gland greatly influences the development of the organs of generation, and is, in its turn, markedly affected by the sexual changes in the life of an individual. This connection, however, appears to be more marked in the female, in whom congestion and functional hypertrophy occur during puberty, menstruation, pregnancy and lactation. (The infantilism and asexuality of cretins is well known.) Associated with this thyroidal swelling may occur

[1] *Am. J. M. Sc.*, 1907, cxxxiv. 390.

fine tremors, sweatings, flushings, nervousness, mental depression, palpitations, some tachycardia, elevation of temperature, loss of weight, exaggerated reflexes and pigmentation. Further, when pregnancy occurs in a myxœdematous woman it is sometimes associated with marked improvement in her condition. Hyperthyroidism and Graves's disease are closely allied, the main difference being one of dosage. Symptoms of hyperthyroidism may occur in patients suffering from simple goitre, thyroid cysts, adenoma, carcinoma, and tuberculosis of the gland. Further, they may be noted when the gland is the seat of venous engorgement, secondary to chronic heart disease. Digestive toxæmia, syphilis, diabetes and Bright's disease, may all be associated with a toxic form of hyperthyroidism. Again, hyperthyroidism may be met with in the early stages of tuberculosis; in fact, the two conditions are frequently confused, having several symptoms in common, viz. slight pyrexia, sweating, flushing, increased reflexes, rapid pulse, and decline in the body weight. Most cases of pronounced chlorosis are also accompanied by manifestations of hyperthyroidism.

The thyroid secretion probably plays a part in the resistance offered by the body to acute infections. Vincent[1] found swelling of the thyroid in 50 per cent. of his cases of acute rheumatism, in 11 of 17 cases of typhoid, in 1 of 4 of meningitis, in 7 of 15 of measles, and so on. It is interesting to observe that many cases of Graves's disease have suffered from preceding acute infections, and possibly there is some connection between the two, for any prolonged functional strain of the gland may give rise to a condition of irritability, which only requires some exciting factor of a sudden functional demand, such as shock or emotion, to establish Graves's disease.

**Hypothyroidism.**[2]—In addition to the three well-known conditions of complete hypothyroidea—myxœdema, idiocy and cretinism—a less well-defined form, due to a partial diminution of the thyroidal secretion, is described under the name of "myxœdema fruste." Many of these cases are said not to tend to progress to complete myxœdema. The symptoms are as follows: There is marked anæmia, with diminution of the red cells and hæmoglobin; the heart is dilated and weak, and the pulse rate is frequently increased, running up to 120. The skin is dry and pale, with supraclavicular pads of fat. Backache, violent joint pains, paræsthesias, neuralgias and pseudo-angina, may all be prominent symptoms. There is marked asthenia, a subnormal temperature and subjective feeling of cold, great dyspnœa, and the characteristic giving way of the knees. Profuse hemorrhages may occur. The facial expression is described as one of "sorrowful fatigue." The hair becomes

[1] *Bull. et Mem. Soc. Méd. de Paris*, 1906, xxiii. 598.
[2] *Am. J. M. Sc.*, 1907, cxxxiv. 859.

prematurely grey, and may become brittle and fall out. Transitory glycosuria has been noted, and albumin and casts may be present. That this condition is due to inefficiency of the thyroid is proved by the facts that there is some atrophy or fibrosis of the gland, that it may be caused by partial thyroidectomy, and that it is relieved by the administration of thryoid.

**Secretory nerves of thyroid.**—Edmunds[1] has carried out a further series of experiments on the question of the existence of secretory nerves to the thyroid, which seem to show that a paralysing operation, which consisted in the removal of a great length of the superior laryngeal and vago-sympathetic nerves on one side of the neck in dogs does have some effect, both clinical and pathological, but that this effect is only slight. He infers that the secretion of the thyroid is mainly due to a chemical stimulus, which acts directly on the secreting cells or on the nerve endings distributed to them, or possibly through the nerve fibres which pass along the blood vessels. In favour of the view of special secretory nerves, he adduces the question of the influence of fright as an exciting cause of Graves's disease.

**Hypoparathyroidism.**—Halsted[2] describes a case of parathyroid insufficiency secondary to operation, in which the condition was recognised and ameliorated by the administration of parathyroid glands. Tetany was not permitted to develop, neither were Trousseau's nor Chvostek's signs obtained. The removal of the parathyroids was accidental, and occurred during the excision of a goitre. The symptoms from the first were peculiarly periodic, coming on in attacks consisting of a feeling of numbness in the lips, as if the circulation of blood had stopped in them and then been suddenly let in again. These feelings passed over the face and down the arms, were said to be "indescribably awful," and were accompanied by inability to close the eyelids. During these attacks the patient looked alarmingly ill. Administration of parathyroid gland produced almost instantaneous and marked improvement : the loss of weight ceased ; the appetite, which had been perverted, improved ; the "attacks" were cured ; but a feeble neurasthenic condition persisted, and eventually the patient became a complete invalid, in a condition of cachexia parathyreopriva. Later, constant and distressing pains were felt in the knees, elbows and shoulders, with much crackling in the knee-joints.

Experimental work[3] on the parathyroid shows that tetany develops with great regularity in dogs after the extirpation of the parathyroids. This tetany can, however, be completely removed for several days by saline infusion into a vein, following venesection. Emulsion of parathyroid, injected into the veins,

1 *J. Path. and Bacteriol.*, 1907–8, xii. 102.

2 *Am. J. M. Sc.*, 1907, cxxxiv. 1.

3 *Bull. Johns Hopkins Hosp.*, 1907, xviii. 327.

has also cured well-developed tetany. Violent tetany, which developed in a girl from whom most of the parathyroid was removed during an operation for goitre, was relieved by subcutaneous injection of an emulsion of the parathyroids of the cow.

The internal secretion[1] of the parathyroid seems to differ in at least one important point from that of the thyroid in the absence of any appreciable amount of iodine in these glands. It is interesting to note[2] that it is very much more difficult to produce tetany in herbivora than in carnivora after parathyroidectomy. This may be connected with the greater toxicity of the food of the latter, but possibly it is due to a wider distribution of parathyroid tissue in herbivora, some of which escapes removal.

Many observers[3] have recently maintained that atrophy of the thymus in children and wasting of the tissues go together, Dudgeon[4] and others stating that atrophy of the thymus is the most constant lesion in marasmus. Glandular atrophy in marasmus is not, however, always confined to the thymus. R. L. Thompson describes a case of infantile marasmus in which extreme atrophy was found in the thyroid, medullary suprarenals and parathyroids, in addition to the atrophy in the thymus. In this case the liver, spleen and kidneys, were practically normal, though there was noticeable atrophy of the intestinal mucosa. The atrophy of the parathyroids in this case led to further research, the condition of the parathyroids in 12 cases of infantile marasmus being compared with the condition of these glands in 12 control cases. The result showed that these glands were only just a little more than half the size of those in the control cases. The histological changes which occur are (*a*) degenerative, (*b*) sclerotic, the former affecting the parenchyma, the latter the interstitial tissue. The author does not imply that the atrophy of the parathyroid is the cause of the marasmus, but that the profound changes which occur in the ductless glands must affect the elaboration of internal secretions, which are so essential to health. These histological findings help us to see more clearly why the results are so frequently fatal.

The conclusions reached by Forsyth[5] as to the nature and functions of the parathyroids differ in many respects from those enumerated above. He holds that they are portions of the main thyroid gland which have not yet formed vesicles, but which are functionally active. Further, he holds that parathyroid tissue may develop into thyroid tissue, and that the secretion of the parathyroid glands is a colloid material indistinguishable from thyroid colloid. The parathyroids therefore, according to this author, possess no peculiar function.

J. R. Charles.

[1] *Ibid.*, p. 331. [2] *Ibid.*, p. 333.
[3] *Am. J. M. Sc.*, 1907, cxxxiv. 562. [4] *J. Path. and Bacteriol.*, 1905, x. 173.
[5] *Quart. J. Med.*, 1907–8, i. No. 2.

